# From ownership to custodianship: rethinking human biospecimens data governance in the Chinese context

**DOI:** 10.3389/fgene.2025.1621739

**Published:** 2025-08-07

**Authors:** Yifan Peng, Jiyuan Li, Ruipeng Lei

**Affiliations:** ^1^ School of Philosophy, Huazhong University of Science and Technology, Wuhan, China; ^2^ School of Marxism, Center for Ethics and Governance of Science and Technology, University of Electronic Science and Technology of China, Chengdu, China

**Keywords:** human biospecimens data, ontological status, moral status, custodianship, data trust

## Abstract

As biomedical research shifts from being experiment-driven to data-driven, the importance of human biomedical data (HBD) has been increasingly highlighted. In this context, traditional data governance models face significant challenges. This paper, based on exploring the ontological and moral status of HBD, analyzes the limitations of the current ownership model and proposes a data governance framework centered on custodianship. The paper then highlights how custodianship aligns with Chinese culture, law, and the concept of “Responsible Research and Innovation (RRI)” through an analysis of current policies in the European Union (EU), the United States (US), and China, while also discussing the application of data trust as a specific implementation mechanism. Finally, the paper points out that transitioning to a custodianship model can more effectively balance privacy protection with innovation, data security with technological development, and data sharing with data protection, offering a new perspective and approach for China’s HBD governance and providing a Chinese solution for global HBD governance, thereby promoting the healthy development of life sciences and technology.

## 1 Introduction

The rapid convergence of contemporary life sciences and big data technologies has fundamentally redefined traditional biobanks, evolving them from experiment-based repositories to data-centric infrastructures. Consequently, Human Biospecimens Data (HBD) has emerged as a novel strategic resource with substantial scientific and commercial value (Fecher et al., 2015). It refers to data derived from human biospecimens, including genetic sequences, multi-omics data, clinical records, and other health-related information, all of which can be systematically collected, stored, analyzed, and shared through digital infrastructures and databases. As a key driver of precision medicine and biomedical research, HBD is increasingly becoming a cornerstone of knowledge generation and technological innovation.

Acknowledging the strategic value of data resources, the Chinese government officially recognized data as a critical production factor—on par with land, capital, labor, and technology—in 2020 (Opinions of the CPC Central Committee and the State Council on Building a More Perfect System and Mechanism for the Market-based Allocation of Factors, 2020). Subsequently, landmark national legislations, including the *Data Security Law* (DSL), and the *Personal Information Protection Law* (PIPL), have established a foundational framework for data governance. Additionally, specialized regulations and law such as the *Interim Measures for the Management of Human Genetic Resources* (IMHGR), the *Regulations on the Management of Human Genetic Resources* (MHGR), and the *Biosecurity Law of the People’s Republic of China* (BSL) have been enacted to refine and supplement governance specifically targeting biological resources. At the same time, international instruments and regional regulations, including the *Convention on Biological Diversity* (CBD) and the *General Data Protection Regulation* (GDPR), provide global benchmarks for the governance of data and biological resources.

Nevertheless, the existing regulatory framework in China reveals significant gaps and challenges when applied to HBD. Current legislation has not sufficiently clarified or addressed the attribution of rights related to HBD, and international or regional policy standards cannot be directly transplanted into the Chinese context without critical adaptation. While it is important for China to draw upon global governance experiences, caution must be exercised to ensure that such frameworks are aligned with local cultural, legal, and social conditions. As a result, ambiguities surrounding the legal entitlements to HBD have led to widespread hesitation among stakeholders, manifesting as reluctance or apprehension towards data utilization, significantly hindering the sharing and exploitation of HBD resources. Traditional academic discourse on HBD has predominantly centered around ownership issues, attempting to determine who exactly owns HBD. However, this ownership-centric approach encounters substantial practical limitations, as granting exclusive rights to any single party fails to address the complex ethical and governance challenges intrinsic to HBD. Consequently, it is crucial to shift from the traditional ownership model to a more inclusive and pragmatic custodianship paradigm, shifting governance discussions from the question of “who owns the data” to “how to protect and responsibly share the data” in an ethically sound and effective manner.

This paper first explores the ontological status of HBD, highlighting its unique characteristics and its intrinsic relationship with the human body. It then examines the moral status of HBD, addressing the appropriate ethical perspectives for data that are both sensitive and of significant public value. The paper subsequently critically evaluates various stakeholders’ claims regarding the ownership of HBD, emphasizing the limitations of traditional ownership-centered frameworks. Finally, based on these analyses, the paper argues that a governance model centered on custodianship would be more suitable and effective in the Chinese context.

## 2 Ontological status of human biospecimens data

The concept of HBD used in this paper encompasses all data derived from the human body, including biological characteristics, genetic information, and health-related data, particularly those obtained through the analysis of biological samples from individuals. Due to its close association with human biospecimens, HBD is inherently more unique compared to ordinary personal data (e.g., phone numbers, shopping information), possessing attributes such as human origin, biometric, non-consumability, familial relevance, non-exclusivity, and stark power asymmetries.

Human origin refers to the fact that HBD is essentially derived from human biospecimens and is closely tied to an individual’s biological characteristics. Unlike behavioral data, such as financial transaction data or social media data, which are indirectly generated by external activities, HBD is directly derived from an individual’s biological information (Lynch, 2017). It is produced without artificial intervention, serving as a true reflection of the human body and being inseparable from the individual’s biological essence, such as DNA, fingerprints, irises, brainwaves, blood composition, etc., (Prainsack and Buyx, 2017). The human origin of HBD is an inherent attribute that establishes a strong and natural correlation between the data subject and the data. This characteristic forms the basis for the strong identity binding of HBD and endows it with special ethical significance: human biospecimens data is not merely an information object but is also regarded as an extension of personality rights. During collection and utilization, we must ensure that individuals are not reduced to mere data resources.

HBD also has the feature of biometric, which is unalterable and unique (Wang, 2023). Each individual’s DNA sequences, fingerprints, iris patterns, facial features, and other HBD are globally unique and cannot be replicated or replaced. This high degree of individualization makes HBD a vital basis for identity authentication and precision medicine. Unlike personal information, such as social media accounts or passwords, which can be modified or updated, HBD becomes permanent once leaked or misused, and data subjects cannot restore security through any changes. Therefore, the biometric feature of HBD poses greater challenges for privacy protection and security risk management than ordinary personal data.

HBD exhibits the characteristic of non-consumability that when used or shared, it is not “consumed” or “depleted” by a single use and can be reused indefinitely, continuing to function in diverse research and application scenarios. This characteristic fundamentally distinguishes HBD from traditional biospecimen resources (such as blood or tissue samples) and other finite resources (like energy or minerals) — it does not deplete with use; rather, it has the potential to generate new scientific value through repeated utilization and multi-dimensional analysis (Leonelli, 2018).

Familial relevance means that HBD belongs not only to individuals but also has inherent genetic connections with families, populations, and even entire species. This characteristic originates from the vertical transmission of genetic and epigenetic information, where genes are passed from parents to offspring through germ cells, forming a natural familial biological information chain.

HBD possesses the characteristic of non-exclusivity, which stems from its replicability and non-consumability. Unlike traditional physical resources, once HBD is digitized and stored, it can be infinitely copied, analyzed, and disseminated without damaging the original data. Rapid technological advancements continuously reinforce this non - exclusivity: while individuals are the providers or donors of data, its storage, analysis, and value extraction rely on various research institutions and platforms, and the subsequent translation of results into practical applications depends on institutions and enterprises. In this process, the value of HBD cannot be realized without collaboration among all parties. The feature of non-exclusivity thus drives the sharing of HBD.

HBD also exhibits stark power asymmetries, first evident in data acquisition. Asymmetry refers to the significant power imbalance between data providers (individuals) and data users (such as biobanks, enterprises, research institutions), where individuals often occupy a relatively vulnerable position. When providing HBD, individuals rarely have a clear and complete understanding of how their data will be used, let alone the details of its sharing, analysis, and post-storage applications. In practice, when donating sample materials or data, individuals typically sign broad informed consent forms, lacking genuine knowledge of how the data will be applied afterward - and thus lacking a full understanding of potential risks (Knoppers et al., 2020). In contrast, data users are typically in a position of advantage, holding control over the data and able to dictate its future trajectory. This asymmetry poses significant challenges in the ethical and legal dimensions of HBD, particularly in managing data privacy, ownership, reuse, and cross-border mobility.

These six characteristics of HBD collectively form the underlying logic of disputes over its ownership. Rooted in the identity-bound uniqueness of HBD, these challenge traditional concepts of ownership due to the data’s infinite replicability and non-consumability. They transmit intergenerational biological imprints of families and ethnic groups, yet exacerbate power imbalances between individuals and research institutions through technological monopolies and high asymmetry. They call for open sharing driven by public research needs (non-exclusivity), while demanding strict controls to protect human dignity and privacy. These characteristics compel society to redefine the “ownership of HBD” and seek governance wisdom that transcends zero-sum games between ethical principles and technical rationality, individual sovereignty and collective collaboration.

## 3 Moral status of human biospecimens data

Moral status is defined as: an entity ought to be treated or not treated in a certain way by virtue of its own nature (Beyleveld and Brownsword, 2001). Regarding moral status, it can be considered from two dimensions: the threshold dimension and the gradable dimension.

The threshold question of moral status refers to the idea that there exists a threshold among entities: those that cross this threshold have moral status, while those that do not lack moral status, without considering differences in the degree of moral status. Exploring the threshold view allows us to determine whether human biospecimens and HBD should be subject to considerations of moral status.

The gradable question of moral status concerns the degree or level of moral status. From a gradable perspective, there are two viewpoints: If an entity has moral status, it possesses equal moral status and should be treated equally. And if an entity has moral status, there are gradations in the moral status it holds, which can be determined based on its distinct characteristics (Luo and Ma, 2022). The first “one-size-fits-all” perspective has obvious flaws due to its absoluteness. In our daily lives, there are many entities such as human corpses, organs, animals, and plants. If we were to grant them exactly the same moral status as “humans,” burning a human corpse would be treated as “manslaughter” - a conclusion that clearly contradicts common sense. Conversely, if we treat them as mere “objects” devoid of any moral status, discarding a corpse would be no different from throwing away a paper ball, which appears overly callous and runs counter to our moral intuition. In short, the first view arbitrarily assumes that humans, human biospecimens, and HBD all possess the same moral value, which is not only overly simplistic but also constitutes an unequal moral consideration in itself (De, 2008). The second perspective avoids falling into the dogmatism of “absolute equality” and “uniform treatment.” Based on the view that moral status has a hierarchical nature, this paper thus explores the gradable question of the moral status of HBD.

The ontological status of HBD provides a direct foundation for its moral status. Given that HBD is both a form of human heritage and can be seen as a special identifier of individuals, it follows that such data cannot be simply equated with humans nor treated as mere objects. We may view it as a special entity existing between humans and objects. But how should we approach this unique entity? Before addressing this question, we must first clarify whether HBD, as a special digital form, should be subject to ethical consideration. If so, what should its moral status entail?

Typically, the moral status of an entity is determined by whether it possesses reason, intrinsic value, consciousness, and the ability to perceive and learn (Warren, 1997). HBD lacks consciousness, emotion, and thought, and thus does not possess the same moral status as humans. In essence, HBD shares no fundamental difference from ordinary data - both are objectively stored in computer systems. The data-level distinctions between humans, objects, animals, and plants are negligible; digitization obliterates the boundary between “humans” and “others,” and “objects” do not have moral status. However, it is crucial to note that, on the one hand, HBD differs from ordinary data: it exists in computer systems through the carrier of human biospecimens, which in turn are the bearers of personal life and integral to human life forms. On the other hand, we must recognize that HBD emerges alongside technology, which itself develops as human subjectivity advances - imbued with a human - centric nature. In a sense, HBD represents an extension of the individual. When confronting such data, we inevitably associate it with human beings themselves. If we disregard or completely “objectify” HBD, over time, this attitude may eventually reflect back on humans themselves, which is morally problematic. Treating HBD indistinguishably from ordinary data risks reducing humans to mere “hard drives” for storing data, challenging human dignity and moral status. Therefore, we should situate the moral status of HBD between “humans” and “objects”, granting it an incomplete moral status - neither fully equivalent to humans nor arbitrarily reducible to objects. However, many entities possess incomplete moral status, and there must be differences among them. For HBD specifically, what are the moral boundaries it ought to have?

HBD is personalized and uniquely individual, possessing partial human moral status and thus deserving our respect. Here, “respect” refers to honoring its inherent humanity - just as we respect our own organs, sperm, or eggs - rather than fully objectifying it as property or a commodity to be exploited for profit at will. Additionally, by regarding HBD as an extension of the individual, we must protect its associated rights: privacy rights, the right to be forgotten, and the right to informed consent. At the same time, HBD can be “utilized” - a term distinct from ordinary “use”. This utilization acknowledges that it can generate legitimate benefits through collection, mining, processing, and analysis. While such utilization cannot entirely divorce itself from profit-making, its purpose is noble: benefiting all of humanity - making condemnation of this activity meaningless. Even if prohibiting its utilization were morally unassailable, in the long run, such excessive moral demands might contradict the interests of moral subjects, undermining their moral legitimacy, imposing undue moral burdens, and clashing with the pursuit of a fulfilling life (Yan, 2020). The fundamental reason humans have morality lies in the recognition that morality helps manage mutual interests and yields mutually beneficial outcomes (Wan, 2002). A comprehensive moral framework will not long conflict with the overall interests of humanity. Granting HBD an excessively high moral status could lead to issues such as imbalances in social fairness and justice due to over - empowerment, while also hindering the progress of life sciences. As a scholar once noted: “We cannot, nor should we, shut the gate on technological advancement. Only romantic fools mutter about returning to a state of nature. Rejecting technology is not only foolish but also immoral” (Alvin Toffler, 1996).

HBD should be regarded not merely as an instrumental resource, but also as an end in itself. Its intrinsic connection to personal identity must be fully recognized and respected, ensuring proper safeguards that prevent technological rationality from overriding fundamental human values. At the same time, it is important to acknowledge the resource dimension of HBD and affirm its substantial potential to generate significant social and economic benefits.

## 4 Ownership issues of human biospecimens data

Building upon the previous exploration of the ontological and moral status of HBD, a central issue arises: to whom should HBD legally and ethically belong? Due to its dual nature as both a deeply personal entity and a valuable resource, ownership disputes over HBD are complex, with no clear consensus among stakeholders. The following chapter will explore the various claims to ownership of HBD made by different stakeholders.

### 4.1 Human biospecimens data belongs to the providers

From the perspective of the ontological status of HBD, many scholars argue that such data should belong to its providers. HBD exists objectively in computer systems through the carrier of human biospecimens, which are part of the human body. Even when separated from the body, these biospecimens and HBD derived from them remain a holistic and inseparable entity over time. Therefore, data providers should be prioritized as rights holders and entitled to exclusive data property rights. Other scholars argue from the non-utilitarian nature of biobanks and the ultimate human-centric goal of medical research. Erik Christensen first posits that all medical research, in some sense, benefits individuals. Although biobank research is seen as a societal resource, its ultimate purpose is to improve individual wellbeing - specifically, that of the data providers. He further contends that we often analogize biobanks to “libraries” and human biospecimens to “gifts,” both of which emphasize individuals’ obligations to donate their biological materials to society. From the standpoint of fairness and justice, however, such obligations must be balanced with individuals’ rights (Christensen, 2009). Finally, based on the previous two arguments, he also proposes the view that HBD should belong to its providers. Some scholars further argue that donated materials, due to the lack of absolute de-identification, fall within the scope of application of the PIPL, thus reverting to the realm of individual rights. Data providers worry that if they do not own their HBD, exercising their right to withdraw consent may encounter resistance from other stakeholders, making it difficult to destroy the associated biospecimens and HBD. From an equality perspective, legally empowering data providers allows them to enjoy various health-related benefits derived from their biospecimens data. This prevents providers from being reduced to victims of capitalist hegemony due to the asymmetry in knowledge and rights, where they might otherwise be exploited under imbalanced power dynamics (Ducournau and Strand, 2009).

### 4.2 Human biospecimens data belongs to the custodians

Some scholars insist that HBD should belong to custodians (often referring to biobanks). They argue that explicitly recognizing a legal right is the most effective means to achieve practical objectives and prevent misuse, as this grants biobanks the authority to protect biospecimens and HBD. Without such recognition, repositories would find it nearly impossible to fulfill their functions: “It creates the impression that people can come back and reclaim their tissue.” Those holding this position often add that providers need only be informed in informed consent documents that biobanks will acquire ownership of the biospecimens and HBD collected from them (Boggio, 2016).

From the functional perspective of biobanks, they serve as a “central hub” for data, storing biospecimens collected from providers, safeguarding HBD, and supplying it to users to maximize its utility. Biobanks thus play a connecting role between upstream providers and downstream users. Assigning ownership of HBD to data custodians (biobanks) facilitates smoother data circulation, reduces unnecessary intermediate procedures, and largely isolates direct contact between data providers and users. This significantly protects providers’ privacy and mitigates risks of discrimination, stigmatization, or other harms.

The stance of assigning ownership of HBD to custodians aligns with providers’ trust. Individuals often participate in biomedical research for altruistic motives, donating their biospecimens and HBD as “gifts”. While this means donors do not retain explicit rights to dictate how their materials are used, the primary driver for their voluntary contribution is trust - they trust that biobanks will properly preserve their biospecimens and HBD, protect their privacy, and ensure these resources benefit society at large. Therefore, assigning ownership to custodians helps maintain providers’ trust and ensures that such altruistic donations can be sustained in a long-term, positive manner.

### 4.3 Human biospecimens data belongs to the users

Those who advocate this stance argue that assigning rights to HBD to either providers or custodians not only ignores the contributions of data users but also overlooks how the value of such data would diminish significantly without user involvement. Users invest substantial human, material, and financial resources into utilizing the HBD. From the perspective of total investment costs, data users often contribute more than providers or custodians, making it reasonable for ownership to lie with users. Some scholars go further, asserting that in the era of big data, the primary value of HBD lies in its collection, recombination, and secondary use (Zhang, 2021). They acknowledge that HBD itself does not inherently justify property rights, but argue that users transform the data into information through research, mining, and other means of “relational processing” - a process that involves value addition and evolves “data” into “big data.” This transformation obscures the uniqueness of the human elements in HBD, serving as the basis for rights empowerment. Crucially, this process demands substantial investments of effort and capital from data users, who argue that such contributions merit ownership claims over the data’s derived value (Su, 2017). The value of HBD consists of two parts: absolute value as an inherent part of the human body, which remains unchanged; and relative value as a “commodity” with context-dependent utility. While isolated HBD has limited standalone value, its true power in advancing precision medicine and human genomics lies in massive datasets. Only through statistical analysis of large-scale HBD can we identify correlative patterns between genetic information and phenotypes, construct genetic maps, and predict hereditary or familial diseases.

From a cost-control perspective, we should support assigning ownership of HBD to users. Using personal data as an example, Wang Zhong argues that if laws grant property rights over personal data to providers, it would incur significant and unnecessary costs (Wang, 2015). Imagine that if providers or custodians are recognized as rights holders of HBD: this would mean data users would need to communicate with providers/custodians for any action involving the data, and service providers would have to negotiate with them for every use. This would not only generate substantial transaction costs but also increase risks of privacy leaks during repeated interactions with providers, potentially leading to unfair issues like discrimination.

From the perspective of the long-term development of big data medicine, assigning ownership of human biospecimens data to users can encourage continued investment, research, and translation of such data by maximizing economic benefits. The *de facto* practice in society today is that HBD belongs to users, and challenging this unwritten rule would inevitably cause industry-wide turbulence. Not all data users are non-utilitarian - businesses are profit-driven. If we fail to protect users’ rights and interests through law, the production of HBD will stall, hindering the development of the entire biopharmaceutical industry and running counter to the purpose of the data rights confirmation system (Su, 2021).

### 4.4 Human biospecimens data belongs to the public

The *Universal Declaration on the Human Genome and Human Rights* states that “genetic information belongs to humanity as a whole”. Adhering to this principle and aiming to promote the health and wellbeing of all humanity, some scholars argue that HBD should belong to the public and be managed by the government.

From a utilitarian perspective, HBD can improve disease cure rates and prioritize enhancing human wellbeing, fully aligning with utilitarianism’s principle of “the greatest happiness” - though this refers not to individual happiness, but to the maximum happiness of all relevant individuals. Therefore, we must consider the happiness of all humanity. These scholars analogize human biobanks to “libraries,” arguing that profit-making or commercialization of sample databases contradicts the original purpose for which biobanks were established: to serve the common good (Lei and Qiu, 2018). By framing human biospecimens as “gifts” and defining biospecimens donation as an act of altruistic giving (Titmuss and Michael, 1999), proponents argue that no party - providers, custodians, or users - should be entitled to claim ownership. This reasoning posits that since donations are not meant to yield personal benefits, none of these groups are suitable as rights holders for HBD. Instead, HBD should belong to the public at large, grounded in the principle that such resources are collective assets contributed for societal benefit rather than private gain.

Another perspective posits that once we recognize humanity’s dependence on society, our obligation to uphold the common good becomes as important as our individual rights to freedom (Cambon-Thomsen, 2004). Human biobanks and HBD constitute a shared, indispensable high-value interest, and realizing this common good inherently fulfills our own existential value. Proponents of this view argue that assigning ownership of HBD to the public not only serves the collective interest but also aligns with the realization of our individual value - rooted in the understanding that societal wellbeing and personal flourishing are inextricably linked.

This stance more closely aligns with the principle of social equality. When there exist goods essential to realizing one’s conception of the good life - regardless of their specific utility for individual life plans - we have an obligation to ensure everyone can access them equally, so that all may have fair opportunities to live their desired lives. HBD is such a good, as it has the potential to vastly improve our future. If ownership were granted to any single rights-holding entity, equal access for all would be impossible. Only by assigning it to the public at large - treating it as a shared commons - can we ensure equitable access. Furthermore, HBD contains shared hereditary material, a form of cultural heritage, making equal access not just a matter of justice but also ethically sound.

## 5 Governance pathways for human biospecimens data in China

### 5.1 Current regulatory framework on HBD

This section provides a critical overview of the current regulatory frameworks governing HBD at both the international and national levels, with a particular focus on China. Through a systematic examination of existing policies, legal and practices, this section aims to elucidate their respective strengths, identify regulatory gaps, and pinpoint areas requiring improvement. Such an analysis will lay a rigorous foundation for exploring more adaptive, ethically sound, and contextually appropriate governance models in the subsequent discussion.

#### 5.1.1 Regulatory framework in Western context

Globally, the United States and Europe lead the way in HBD governance. In the U.S., governance is primarily shaped by sector-specific legislation, with the *Health Insurance Portability and Accountability Act* (HIPAA) serving as the cornerstone. HIPAA establishes rules and protection standards for *Protected Health Information* (PHI) and other sensitive data. However, HIPAA is domain-specific, applying only to healthcare entities and a narrow category of businesses contracted with these entities, rather than to all individuals and companies handling healthcare data. The law focuses on safeguarding individuals’ rights to access, privacy, and informed consent (Boyne, 2018). The *Health Information Technology for Economic and Clinical Health Act* (HITECH) supplements HIPAA by strengthening security requirements for personal health information in electronic records and increasing penalties for violations. The *Genetic Information Nondiscrimination Act* (GINA) primarily prohibits employers and health insurance companies from making discriminatory decisions based on personal genetic information. The *Medical Ethics and Regulations* (MER) emphasizes the confidentiality of medical records and the protection of personal privacy. The *Personal Privacy Act* (PPA) aims to safeguard individuals’ sensitive information from unauthorized access and misuse. While these laws focus on privacy and security protection, they do not address issues of ownership. The U.S. *Patent Law* does not specifically regulate genetic data within HBD, but technologies, methods, and innovations in the field of genetics can be patented if they meet the requirements set forth by patent law. Overall, the strength of the U.S. model lies in its well-established healthcare privacy regulations; however, its limitations are evident, particularly due to its narrow scope, which is limited to the healthcare sector. As digital technologies continue to advance, various actors outside the healthcare field are increasingly recognizing HBD as a valuable resource (Terry, 2017), and the privacy and security protections under the existing HIPPA framework may no longer suffice (Oakley, 2023). More importantly, the U.S. has yet to enact a unified federal law to protect HBD, lacking a comprehensive and systematic legal framework to address the increasingly complex issues of ownership and privacy.

European Union is recognized for its comprehensive data protection framework, with HBD governance primarily based on the GDPR. The GDPR categorizes HBD as a special type of personal data, generally prohibiting its processing unless specific legal grounds are met. It grants data subjects essential rights, including the right to be informed, the right of access, the right to rectification, the right to restrict processing, the right to data portability, and, in certain circumstances, the right to erasure (the “right to be forgotten”)(Regulation, 2018). In recent years, the EU has acknowledged the need to further facilitate HBD sharing within the scope of GDPR. To this end, the *Data Governance Act* (DGA) was introduced to promote data sharing and utilization, alongside efforts to establish the *European Health Data Space* (EHDS), supported by the *European Health Data Space Regulation*. Regarding the ownership of HBD, the EU does not regard personal data as tradable private property, but views it as an extension of personal identity, emphasizing individuals’ fundamental rights and control over their data. Rather than “owning” the data, individuals are understood to have control over the disposition of their data. Overall, the EU ensures that individuals can assert their rights over HBD in all situations, with minimal risk of abuse. However, the EU model faces challenges, notably the potential overemphasis on individual control, which may restrict data sharing and hinder research (Peloquin, 2020).

#### 5.1.2 Regulatory framework in China

China’s current data protection policies are, to some extent, influenced by the GDPR, yet they also include distinct provisions and differences. Notably, the GDPR does not explicitly differentiate between “personal data” and “personal information,” whereas, in China’s legal framework, these terms are fundamentally distinct. In Chinese law, “personal information” primarily focuses on the protection of personal rights, with an emphasis on the privacy aspect of the data, while “personal data” is more concerned with the technical characteristics, such as compliance with legal requirements regarding data collection, storage, and use (Meng and Lu, 2025). This distinction is crucial for determining whether the data has private attributes and whether it meets the criteria for application under the PIPL. The HBD discussed in this paper not only raises privacy concerns but also involves issues related to public health and social welfare. Therefore, the governance of HBD is not solely dependent on the legal framework protecting personal information. Relevant legal frameworks for HBD governance have already been established, covering two key areas: genetic data and non-genetic health data. addressing aspects such as privacy, security, and national sovereignty. According to the *Data Security Technology - Rules for Data Classification and Grading* (GB/T 43,697-2024), HBD is categorized into two groups: genetic data and non-genetic health data. Genetic data includes DNA sequences, genotype information, and familial genetic traits, all of which directly contain personal and familial identity information and are considered national strategic resources. This data is primarily governed by the IMHGR, the MHGR, and the *Biosecurity Law of the People’s Republic of China* (BL). The IMHGR establish a reporting system for genetic resources, emphasizing privacy and confidentiality (Interim Measures for the Administration of Human Genetic Resources, 1998). The MHGR focus on safeguarding China’s human genetic resources, further clarifying the national sovereignty over genetic data. These regulations require all activities related to the collection, use, and cross-border transfer of genetic data to undergo stringent approval and security assessments to ensure national biological security and public health. Additionally, they support the responsible use of human genetic resources to advance scientific research, foster biomedicine development, and enhance biosecurity and public health (Regulations of the People’s Republic of China on the Administration of Human Genetic Resources, 2019). The BL asserts national sovereignty over China’s human genetic and biological resources, emphasizing secure management, particularly of genetic resources, and provides legal protection for cross-border data flow, international research collaboration, and emergency response (Biosafety Law, 2021). Non-genetic health data, such as metabolic indicators in blood, protein expression profiles, and imaging data (e.g., CT, MRI), are not covered by the MHGR, but are instead regulated by the PIPL and the DSL. According to Article 28, Clause 2 of PIPL, non-genetic health data is classified as sensitive personal information, requiring explicit consent and security assessments during data collection, processing, and cross-border transfer. PIPL consolidates previously fragmented regulations from various administrative laws, creating a more cohesive data protection legal framework in China (He, 2022). Although the DSL does not specifically address non-genetic health data, it rigorously regulates the security and usage of such data through classification management, cross-border assessments, and privacy protection.

However, current regulations still exhibit significant shortcomings in several key areas. First, existing laws do not provide clear provisions on the ownership of HBD. Although Article 127 of the *Civil Code of the People’s Republic of China* affirms that “if the law provides for the protection of data and virtual property on the internet, such provisions shall apply,” it fails to establish a unified and direct regulation on data ownership. The existing legal framework primarily focuses on personal information and privacy protection. For example, PIPL grants individuals rights to access, copy, rectify, and delete their personal data (Personal Information Protection Law of the People’s Republic of China, 2021). However, the framework largely avoids providing an absolute definition of “data ownership,” instead breaking it down into various stages and allowing different entities to exercise “holding rights, processing and usage rights, and product operating rights,” thereby encouraging the marketization of data. The policy document “*The Data Twenty Measures*” proposes a strategy of “innovating data property rights, downplaying ownership, emphasizing usage rights, and focusing on the circulation of data usage rights.” (Li, 2024) The *Science and Technology Progress Law* regulates the open sharing of scientific research data and related legal responsibilities, but does not address data ownership. China has yet to establish a clear and unified legislative framework regarding the ownership of medical data and big data. Moreover, the model of individual control may not be well-suited for HBD (Sun, 2023). Under the PIPL, informed consent is considered a fundamental principle for sharing or processing personal sensitive information. However, HBD is deeply connected to familial identity, which can lead to conflicts between individual consent and collective consensus. The inherent uncertainty of scientific research, coupled with changes in research scope, makes obtaining clear and specific consent more challenging. Additionally, the long-term nature of many studies, along with evolving research content, further increases the complexity and cost of obtaining consent. While de-identified data may fall outside the scope of PIPL, scientific research still requires retaining certain identifiable characteristics, meaning that de-identification protocols may not fully resolve privacy concerns in health data research (Shi, 2024). Furthermore, certain classification and grading standards may lack adequate scientific rigor. In real-world applications, genetic and non-genetic health data are often collected simultaneously. For example, *genome-wide association studies* (GWAS) typically require the analysis of both DNA sequences and electronic health records. In these cases, genetic data is governed by the MHGR, while non-genetic health data falls under the jurisdiction of PIPL and the DSL, with cross-border data transfers requiring security assessments. Due to the involvement of different regulatory bodies and legal frameworks, this cross-departmental governance structure can lead to duplicated compliance efforts, inconsistent standards, and increased compliance costs for research institutions, which may also create barriers to international cooperation.

Overall, the analysis in Section 3 of traditional ownership and in Section 4 of national legal frameworks clearly demonstrates that disputes over HBD ownership have been persistent, and existing legal frameworks are insufficient to resolve these conflicts. These traditional frameworks focus primarily on personal data protection and privacy rights, where individuals may seek to influence complex decisions regarding the sharing and use of HBD in ways that prioritize their own interests. To some extent, this model of individual control restricts data flow (Yassin, 2016). As the future of data-driven governance continues to evolve, the concept of “custodianship” rather than “ownership” is emerging as a potentially more effective governance model. This shift aims to address the imbalance between data subjects and data controllers, ensuring that data use both respects individual interests and benefits society as a whole. The next section will further examine the applicability of “custodianship” and its specific governance mechanisms in China.

### 5.2 Custodianship, not ownership

The mainstream perspective in global data governance has shifted from the early ownership framework to the custodianship framework, with data trust methods being increasingly applied in practice. Custodianship can be defined as the “caretaking responsibility for HBD that starts at the planning of a biobank initiative, prior to the collection, and continues through research use to final dissemination of research results” (Verlinden, 2016). Custodianship emphasizes a management model centered on responsibility, ensuring the appropriate use and protection of data, which stands in contrast to the ownership approach, which treats HBD as property with exclusive rights and control, fully belonging to the owner. In this context, the concept of trust naturally emerges as an extension. The core principle of trust is that the trustee, who is entrusted with managing HBD, must uphold fiduciary duties such as loyalty and diligence on behalf of the settlor. This implies that the trustee must adhere to the “highest standard of honor,” demonstrating self-discipline that exceeds commercial ethics (Bainbridge, 2014). In practice, this means that individuals entrust their HBD to independent third-party organizations for management, ensuring privacy while enabling the reasonable use of the data. As the MIT Technology Review pointed out when considering data trust as a groundbreaking technology: the reason for frequent data breaches is the long-standing reliance on an individual privacy model, where individuals are primarily responsible for safeguarding their privacy. Data trust offers an alternative, representing the interests of individuals by facilitating the collection and management of personal data (Breakthrough Technologies, 2021). Scholars in both the U.S. and the UK have explored governance models based on custodianship and implemented through data trust; however, the operational models differ due to legal system variations. In the U.S., the model is one of “information trustees,” where data controllers are required to assume fiduciary duties, but this remains largely theoretical. In contrast, the UK employs a bottom-up data trust approach, establishing third-party organizations to provide independent data trust services. Given the similarities between the legal frameworks of the UK and China, the UK’s bottom-up model may better suit China’s needs in data protection, privacy preservation, and data sharing (Zhai, 2021).

Blockchain technology has emerged as a critical enabler for implementing custodianship-based health data governance frameworks, offering technical infrastructures that enhance transparency, traceability, and rule enforcement (Yang et al., 2020). In recent years, a growing number of initiatives worldwide have begun exploring the application of blockchain in the governance and exchange of health-related data. Among these, the Decentralized Science (DeSci) movement has gained significant traction. DeSci aims to establish a transparent, verifiable, community-driven, and incentive-compatible ecosystem for scientific data by leveraging decentralized technologies such as blockchain (Weidener and Spreckelsen, 2024). A prominent example of this approach is AminoChain, a representative platform that integrates DeSci principles into the management of health data. By utilizing Layer 2 blockchain solutions, AminoChain has developed a decentralized “biobank” that enables transparent and auditable management of biospecimens and HBD, supporting both clinical and research applications (Gate.io, 2024; Sanchez et al., 2025). Unlike traditional data marketplaces, which often treat data as proprietary assets owned by a single entity, AminoChain adopts a custodianship-based model that emphasizes responsible, trust-based data stewardship across the entire data lifecycle. Through smart contracts, the platform automates access permissions, enforces usage policies, and records all data transactions immutably on-chain, thereby embedding fiduciary obligations into the technical layer.

What distinguishes AminoChain from other international initiatives is its explicit effort to bridge technological decentralization with normative governance responsibilities. For instance, in the U.S., the concept of the“information fiduciary” has been introduced to assign ethical and legal obligations to data controllers. However, this remains largely theoretical and lacks concrete implementation mechanisms. In contrast, the EU has piloted several data trust models that provide third-party stewardship of data, but these are still institutionally governed and rely on manual oversight, with relatively limited technological automation. The exploration of AminoChain illustrates how blockchain can function as a robust operational substrate for data trusts, particularly in contexts requiring cross-border data sharing and compliance transparency. In China, analogous cases are relatively rare. The Gui’an Data Exchange, for example, applies a digital trust framework (First Compliant Personal Data Transfer Transaction Completed, 2023), but it is primarily focused on general personal data and does not address the unique challenges of HBD governance. Similarly, the China Metabolic Analytics Project (ChinaMAP), which opened access in November 2024 to nearly two decades of biospecimens and HBD from over three million individuals, represents a state-led, centralized model of data sharing. While it contributes significantly to biomedical research, it does not reflect the normative or structural features of a custodian- or trust-based model. In this light, AminoChain provides not only a technologically grounded realization of the “custodianship + trust”paradigm, but also offers valuable insights for jurisdictions such as China seeking to develop HBD governance models that align with both local institutional logics and emerging global standards.

In the context of safeguarding individual rights, further promoting the sharing and utilization of HBD through a governance framework centered on custodianship, with data trust as the implementation method, is undoubtedly more suited to China’s needs. The reasons for this are as follows:

(1) It aligns closely with China’s legal and policy frameworks. First, the emphasis on informed consent, privacy protection, and custodianship in the PIPL and DSL corresponds with the core principles of custodianship, which asserts that control over data belongs to the data subject. The flow and use of data must be strictly authorized and managed by trusted third-party entities. The MHGR and the BL both emphasize the national resource nature of HBD, and the government’s responsibility for managing genetic resources can be realized through the custodianship model. Second, data trust can sidestep the issue of ownership by establishing data trusts for personal HBD, while utilizing third-party management and privacy-preserving technologies, such as privacy computation, to ensure data privacy and security throughout the data transfer process. This approach aligns well with the concept of “data property rights division” introduced in the *Data Twenty Measures.*
(2) The Responsible Research Innovation (RRI) concept provides a governance pathway for China. RRI has gained wider importance in Europe in recent years, being part of the European Framework Programmes (e.g., Horizon 2020) as well as discussed and developed in academic publications and European-level projects (Burget et al., 2017). Von Schomberg defined RRI as “a design strategy which drives innovation and gives some ‘steer’ towards achieving societal desirable goals” (Von Schomberg, 2013). RRI emphasizes that innovation should not only focus on technological advancement but also take into account social responsibility, ethical standards, and public interest. Additionally, RRI evokes a collective duty of care (Owen et al., 2020). This aligns perfectly with the shift from ownership to custodianship, which no longer centers solely on the individual but places more emphasis on the collective. Under the custodianship framework, data managers (such as trust institutions) assume responsibility for the data, ensuring its use does not violate the data subject’s wishes, while the control over the data remains with the individual. Moreover, the data trust model, which involves the establishment of independent third-party trust organizations to manage data, inherently offers high transparency and public oversight. However, when discussing RRI, it is important to also recognize the serious consequences that irresponsible research and data practices can lead to. The Facebook-Cambridge Analytica scandal, which erupted in 2018, serves as an example of irresponsible research. Cambridge Analytica exploited user data collected by Facebook for political advertising targeting, manipulating voter sentiment and behavior without explicit user consent, leading to widespread privacy violations. In response, the public launched the DeleteFacebook campaign to address the data privacy breach, aiming to boycott Facebook (Facebook-Cambridge Analytica data scandal, 2025). This incident not only violated user privacy but also had a profound impact on social democracy and public interests. More importantly, it eroded public trust, highlighting the potential for companies to exploit big data collection and analysis for unethical commercial purposes. In contrast, the RRI concept offers a sustainable framework for data governance. By adopting the custodianship framework and data trust model, these issues can be effectively mitigated, providing China with a data governance approach that is both responsible and innovative, while advancing the sharing of HBD.
(3) It aligns with China’s cultural philosophy. Chinese cultural traditions emphasize the mutual responsibility between individuals and society, focusing on balancing social interests and long-term development, which provides a solid cultural foundation for data governance. In the Chinese ideological system, the legitimacy of governance largely derives from the trust and delegation of power by the people. As early as the pre-Qin period, Confucianism emphasized the concept of people-centered governance: “The people are the most important, followed by the state, and the ruler is the least important.” (Mencius, Jinxin). This statement underscores the fundamental role of the people in governance, with the ruler’s authority ultimately coming from the people’s trust. Dong Zhongshu further developed the notion of “the divine right of kings, and power entrusted by the people,” viewing the ruler’s power as a trust granted by heaven and the people. Since the ruler’s authority is a trust from both heaven and the people, the ruler must follow natural laws and serve the people, or risk losing legitimacy (Feng, 2010). The saying “Water can carry a boat; it can also capsize it” vividly illustrates the relationship between royal authority and the people’s trust: the people are like water, and the ruler is like a boat; water can support the boat, but it can also overturn it. The ruler’s power stems from the people’s trust, and only through benevolent governance and moral conduct can the ruler maintain the support of the people. Conversely, if the ruler loses the people’s support, the regime can be overthrown at any time (Li, 2019). In light of this traditional view, custodianship should also be seen as a responsibility entrusted by data providers, requiring adherence to virtue and integrity—principles that align with the core values of trust, such as loyalty and diligence. In conclusion, these cultural concepts provide a deep foundation for custodianship-based data governance in China.

Although custodianship and the data trust framework have demonstrated significant potential for data governance both in China and globally, they also face several practical challenges. The first issue concerns long-term sustainability. The custodianship model requires trust institutions to manage and protect HBD over extended periods. However, if a third-party institution encounters problems—such as bankruptcy, loss of credibility, reduced management capacity, or an inability to continue fulfilling fiduciary duties—HBD security may no longer be guaranteed (Yassin, 2016). The second issue is the lack of uniform standards and regulations. The custodianship model requires the assurance of privacy and security during the global transfer of HBD, yet differing data protection laws and standards across countries and regions make it difficult to ensure that parties in other areas adhere to national standards. Lastly, this custodianship framework may introduce further complications. For example, who is authorized to establish third-party entities? Can custodians charge access fees? If not, how will these third-party institutions remain operational? If they can, might this lead to unfair practices? Bart van der Sloot argues that such a framework could potentially become a “Pandora’s box” for data processing, as data companies might exploit this opportunity to develop new methods of collecting data beyond current practices (Van der Sloot and Keymolen, 2022).

## 6 Conclusion

The governance of HBD is at a pivotal crossroads. On one hand, the knowledge and value embedded in data have never been greater, driving rapid advancements in medicine and life sciences. On the other hand, uncertainties surrounding data ownership and the allocation of rights have raised complex legal and ethical issues. The traditional ownership-centric approach fails to adequately address the current challenges, and may even deepen the divides between stakeholders, rendering HBD unusable or untrustworthy, leading to a deadlock. Custodianship and data trust offer an innovative governance framework for HBD, breaking away from the conventional “ownership” model and offering a potential solution to the impasse. Custodianship focuses on responsible data management, ensuring the protection of data subject rights while facilitating the compliant and secure sharing and use of data. Data trust, as a specific method of implementing custodianship, involves the management of data by third-party trust institutions, ensuring transparency and fairness in data transfer. This framework aligns well with China’s legal system, cultural values, and the principles of RRI. While this model holds considerable potential, it also faces challenges such as ensuring long-term sustainability and consistency in standards. To ensure its successful implementation, further improvements in regulatory mechanisms and technological safeguards are needed, along with enhanced public trust in data governance.

## Data Availability

The original contributions presented in the study are included in the article/supplementary material, further inquiries can be directed to the corresponding author.
